# A Peculiar Case of Pneumonia due to *Mycoplasma pneumoniae* in a Child with Cystic Fibrosis and Sensibilization to *Aspergillus fumigatus*

**DOI:** 10.3390/pathogens9010015

**Published:** 2019-12-22

**Authors:** Laura Peccini, Serena Pennoni, Valeria Mencarini, Marco Saponara, Nicola Palladino, Nicola Principi, Guido Pennoni, Susanna Esposito

**Affiliations:** 1 Pediatric Clinic, Department of Surgical and Biomedical Sciences, Università degli Studi di Perugia, 06132 Perugia, Italy; laurapec91@gmail.com (L.P.); serepennoni@hotmail.it (S.P.); marcosaponara@hotmail.it (M.S.); 2 Cystic Fibrosis Center of Umbria Region, Branca Hospital, 06020 Branca, Italy; valeria.mencarini@uslumbria1.it (V.M.); nicola.palladino@uslumbria1.it (N.P.); guido.pennoni@uslumbria1.it (G.P.); 3 Università degli Studi di Milano, 20122 Milan, Italy; nicola.principi@unimi.it; 4 Pediatric Clinic Pietro Barilla Children’s Hospital Department of Medicine and Surgery, Università di Parma Via Gramsci 14, 43126 Parma, Italy

**Keywords:** allergic bronchopulmonary aspergillosis, antimicrobial resistance, *Aspergillus fumigatus*, cystic fibrosis, pneumonia

## Abstract

*Aspergillus fumigatus* plays a major role in pulmonary exacerbations in patients with cystic fibrosis. The most common *A. fumigatus* diseases are those based on immune-mediated response to *A. fumigatus* antigens; including allergic bronchopulmonary aspergillosis (ABPA). In this condition; the presence of *A. fumigatus* in the lower respiratory tract triggers an IgE-mediated hypersensitivity response that causes airway inflammation; bronchospasms; and bronchiectasis. This case report describes a ten-year-old male patient suffering from cystic fibrosis (CF) in whom the diagnosis of ABPA occurred in association with pneumonia due to *Mycoplasma pneumoniae* more than two weeks after hospitalization. This case is a good example of how difficult the identification of ABPA in CF patients can be and highlights that ABPA can occur in association with co-infections due to other pathogens. In order to avoid the risk of a late ABPA diagnosis, it is imperative that the diagnostic criteria guidelines are reviewed and standardized.

## 1. Background

It has been known for many years that in patients with cystic fibrosis (CF), respiratory bacterial infections, mainly due to *Staphylococcus aureus* and *Pseudomonas aeruginosa*, are extremely common. They are considered the main cause of lung destruction and the progressive reduction in pulmonary function that characterizes this disease [1]. *Mycoplasma pneumoniae* has been rarely reported in patients with CF, although it has been associated with cardiac complications in some cases [2]. Moreover, as recent studies have shown [3], other infectious agents, such as nontuberculous mycobacteria and fungi, can significantly contribute to lung deterioration. Among fungi, *Aspergillus fumigatus* plays a major role [4]. Although rare, this infectious agent can overcome lung defenses and cause invasive disease. Moreover, it can be associated with the development of an aspergilloma. However, the most common *A. fumigatus* diseases are those based on immune-mediated response to *A. fumigatus* antigens, including allergic bronchopulmonary aspergillosis (ABPA) [5]. In this condition, the presence of *A. fumigatus* in the lower respiratory tract triggers an IgE-mediated hypersensitivity response that causes airway inflammation, bronchospasms, and bronchiectasis.

ABPA usually presents as acute or subacute deterioration of lung function associated with evidence of new infiltrates on chest X-ray findings. Sometime, bronchospasms are present. In CF patients, ABPA is not a rare disease; in most studies, it was reported that it was diagnosed in approximately 10% of cases [6,7]. Despite its relative frequency, the significant clinical manifestations and the characteristic laboratory findings, ABPA is frequently underdiagnosed, and diagnosis is frequently made several months or years after the onset of signs and symptoms. This is because the most common and recurrent bacterial infections have overlapping clinical, radiographic, and laboratory features, and differentiation is difficult [8]. Moreover, as reported below, laboratory tests for *A. fumigatus* do not help with a diagnosis. In CF patients, asymptomatic colonization by *A. fumigatus* is relatively common. Skin prick testing or antibody level evaluation may reflect sensitization and not disease. The presence of galactomannan, a marker of active fungal growth, in CF sputum is common in patients with ABPA, but it can also be found in subjects infected by other fungi [9].

To reach an ABPA diagnosis, specific criteria have been formulated by several experts [10]. However, the diagnosis of ABPA remains a diagnosis of exclusion; signs of clinical and radiological lung deterioration not attributable to other causes in association with an *A. fumigatus*-specific IgE or IgG immune response must be demonstrated. The diagnosis of ABPA remains challenging. Overdiagnosis with subsequent steroid treatment or underdiagnosis without treatment leading to clinical deterioration can occur. The case described here is a good example of how difficult the identification of ABPA in CF patients can be, especially if associated with a co-infection due to another pathogen.

## 2. Case Report

A ten-year-old male patient suffering from CF was brought to the Emergency Department of our hospital because of low-grade fever (axillary temperature [AT] 38.0 °C) associated with left hemithorax pain and slight polypnea. In his medical history, several previous recurrences of respiratory bacterial infections were reported. The last episode had occurred approximately 3 months previously, was due to a methicillin-sensitive *S. aureus* and was treated with a combination of amoxicillin/clavulanic acid for 15 days. During the following period, no antibiotic treatment was administered.

At admission, his physical and neurodevelopmental growth was within the normal range. However, he was febrile (AT 39.2 °C), his respiratory rate was 40 breaths/min, his heart rate was 111 beats/min, his oxygen saturation in room air was 91%, and his blood pressure was 114/68 mmHg. Thoracic auscultation revealed cracking rattles in the left basal area and forced expiratory volume in 1 s (FEV1) was 46%. Initial laboratory tests showed leucocytosis with neutrophilia but without eosinophilia (white blood cell [WBC] count 17,130/mm^3^; neutrophils [N] 12,916/mm^3^, 75.4%; eosinophils [E] 462/ mm^3^, 2.7%), an increased C-reactive protein [CRP] serum level (11.19 mg/dL), a high cold agglutinin serum concentration (> 1:16) and IgM and IgG positive for *M. pneumoniae* (specific IgM, 1:300; specific IgG, 1:800). The sputum culture test was negative for respiratory bacteria and fungi. The chest X-ray showed an average left basal thickening, right paracardial thickening and widespread interstitial thickening. Suspecting a bacterial respiratory exacerbation, intravenous therapy with cefazolin (150 mg/kg/day in 3 doses) and tobramycin (10 mg/kg/day in single dose) was started. Moreover, oral clarithromycin (15 mg/kg/day in 2 doses) was added.

Treatment was apparently effective, as clinical conditions rapidly improved and fever disappeared within two days. However, after six days, despite antibiotic therapy being continuously administered, a new episode of thoracic pain associated with the reappearance of fever (AT 38.5 °C) occurred. A second chest X-ray showed a significant increase in the previously identified lung thickening associated with pleural effusion in the left basal site; new blood tests showed an increase of leucocytosis with neutrophilia (WBC 19,870/mm^3^, N 76%), in this case with eosinophilia (E 2384/mm^3^, 12%), and a further increase in the CRP serum concentration (16.80 mg/dL). Suspecting a lung infection resistant to first-line antibiotic therapy, the ongoing drugs, except for the macrolide, were withdrawn, and a new therapeutic course with vancomycin (45 mg/kg/day in 3 doses) and meropenem (60 mg/kg/die in 3 doses) was started.

Contrary to what was observed a few days before, a new sputum culture test was positive for *A. fumigatus* and remained negative for aerobic bacteria. Unfortunately, the new drug regimen was ineffective, as no modifications in clinical conditions and chest X-ray findings were demonstrated after one week of treatment. Moreover, blood tests revealed an increase in the number of WBCs (21,220/mm^3^), N (15,193/mm^3^), and E (1782/mm^3^). CRP was only slightly reduced (9.71 mg/dL).

**Figure 1:**
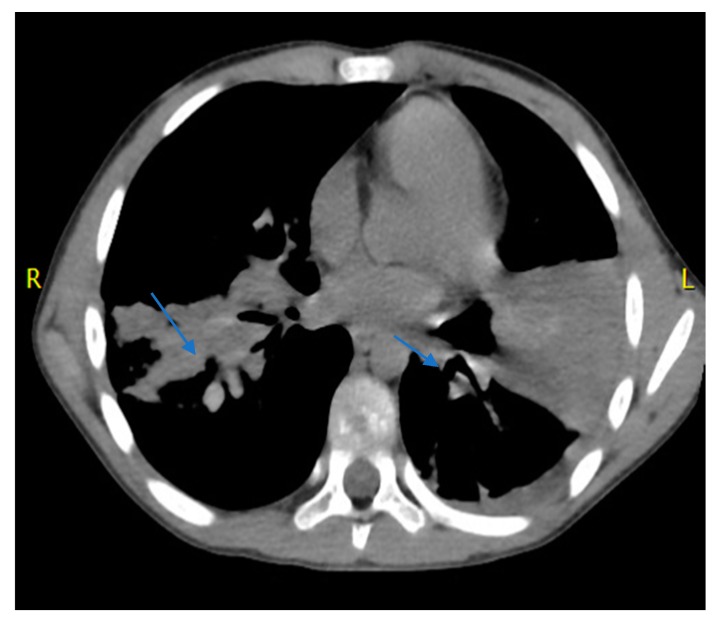
Computed tomography of bilateral bronchiectasis/bronchiolectasis with material in the context, suggesting high-attenuation mucus plugs.

Suspecting an infection due to macrolide-resistant atypical bacteria, clarithromycin was withdrawn, and ciprofloxacin (1 g/day in 2 doses) was started. Three days later, a chest computed tomography (CT) scan confirmed the chest X-ray findings but revealed bilateral bronchiectasis/bronchiolectasis with material in the context, suggesting high-attenuation mucus plugs (HAM) (Figure 1). The blood test showed a reduction in WBCs (17,080/ mm^3^) and N (10,128/mm^3^, 59.3%), but E were found to be significantly increased (3296/mm^3^, 19%). CRP was reduced (4.26 mg/dL).

Considering all these findings, ABPA associated with macrolide-resistant atypical bacterial infection was considered. A bronchoalveolar lavage (BAL) and several tests for the diagnosis of sensitization to *A. fumigatus* were planned. BAL showed spores and fungal hyphae of *A. fumigatus* and was positive for galactomannan. Total IgE, previously undetermined, were found significantly increased (4096 KU/L, n.v. < 100 U/mL). RAST for food and inhalants showed multi-sensitization. Finally, high serum level of anti-*Aspergillus* IgE (26.20 KU/L, n.v. < 0.1 KU/L) was evidenced. However, the galactomannan antigen was negative. A diagnosis of ABPA was made, and endovenous prednisone (1 mg/kg/day) associated with itraconazole (5 mg/kg/day) in 2 doses was started.

**Figure 2:**
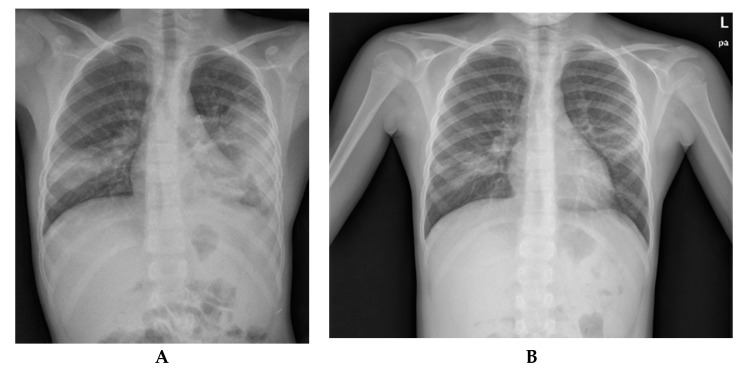
Chest X-ray evolution before allergic bronchopulmonary aspergillosis (ABPA) diagnosis (**A**) and one month after treatment (**B**).

Subsequent blood tests showed a progressive reduction in the flogosis indexes, finally reaching negative CRP and a progressive reduction in eosinophils, while the chest X-ray examination revealed a reduction in lung thickening and the disappearance of pleural effusion. Table 1 summarizes laboratory exams at admission and during follow-up. The follow-up at one month after discharge showed also normalization of radiographic findings (Figure 2), further supporting the diagnosis of ABPA.

The management of this patient was approved by the Ethics Committee of Umbria Region (PED- 2019-9), and both parents provided written informed consent for the evaluation of the child. The Ethics Committee of Umbria Region approved the publication of this case and both parents provided written informed consent for the publication of this manuscript.

## 3. Discussion

This case shows why the diagnosis of ABPA in CF patients can be very difficult and made later than desired. This disease was described in 1952 and largely studied later, with standardization of the key diagnostic features [10,11]. Despite this, no single test is able to definitively establish a diagnosis in all patients with ABPA. The presentation of central bronchiectasis with normal tapering bronchi or of HAM plugs on high-resolution CT is considered strongly indicative or pathognomonic of ABPA [12]. However, these respiratory tract alterations can be observed in only a minority of patients, as bronchiectasis generally occurs in the most advanced stages of illness, and HAM plugs have been found in only 28% of patients with definitively established ABPA.

A set of criteria is required for diagnosis, but no consensus exists on the list of clinical and laboratory findings required to make a definitive diagnosis. This explains why several different criteria have been proposed and diagnosis of ABPA is frequently made when no other explanation for acute lung disease or progressive lung function deterioration is possible. Respiratory problems directly due to CF make the diagnosis of ABPA even more difficult. Bacterial infections are extremely common and cause lung deterioration and remodeling quite similar to those associated with ABPA [5]. Moreover, co-infections are frequent in CF patients and specific signs as well as symptoms are not pathognomonic of a definitive etiology [13,14]. On the other hand, *M. pneumoniae* is not frequently associated with pulmonary exacerbation in patients with CF [2] and recent studies have shown that it presents with clinical, laboratory and radiographic findings similar to those due to typical bacterial infections [15,16].

The case reported herein well exemplifies all the difficulties that can hinder an early diagnosis of ABPA. In a child with CF with a history of repeated respiratory infections, the evidence of a pulmonary exacerbation cannot fail to lead to a suspicion of bacterial infection and first line antibiotics effective against the pathogens commonly found in CF patients should be prescribed. This seems even more reasonable if, as in the case reported here, laboratory tests seem to indicate a bacterial infection and some of the features usually positive in ABPA, such as eosinophilia and sputum culture for *A. fumigatus*, are negative. A poor response to initial antibiotic therapy can lead to the presence of uncommon and/or antibiotic-resistant pathogens and the prescription of different antibiotics, but if these do not modify the disease course, ABPA has to be supposed and then adequate laboratory and radiological tests planned in order to confirm diagnosis and the most effective therapy prescribed in order to reduce risks of further lung deterioration.

In the case reported here, several findings agreed with the criteria for the diagnosis of ABPA suggested by the Cystic Fibrosis Foundation [17]. Together with the acute clinical deterioration, eosinophilia, a serum total IgE level > 2400 ng/mL, and high *A. fumigatus*-specific serum IgE levels were detected. This strongly supports the diagnosis of ABPA, which seemed to be confirmed by the evidence of *A. fumigatus* in the BAL, the presence of HAM on CT and the very fast and complete response to corticosteroid and itraconazole.

Treatment of ABPA is essential to avoid the risk of permanent alterations due to *A. fumigatus*, which can become permanent and significantly worsen the already poor prognosis of CF patients. Treatment is based on corticosteroid use, and the addition of antifungal drugs such as azoles is debatable. Some authors, fearing drug adverse events, suggest the use of azoles only in patients who do not respond to steroid administration, relapse during drug tapering, become steroid dependent, or develop steroid toxicity [18]. Some other experts tend to be more permissive and suggest combination therapy. Their suggestion is based on the results of several uncontrolled open-label case studies that seem to indicate that the administration of azoles in CF patients colonized by *A. fumigatus* and with the deterioration of lung function were associated with improvements in respiratory symptoms and decreases in CT abnormalities [19,20,21]. We choose to administer itraconazole. Treatment was well tolerated, and no adverse events were observed. In the case of CF patients at high risk for developing invasive aspergillosis (for example, those who have had lung transplant), long- term antifungal prophylaxis should be recommended [22]. However, there is not a clear consensus on how to prevent *A. fumigatus* infection in these patients.

## 4. Conclusions

This case report further highlights that to avoid the risk of a late ABPA diagnosis, it is imperative that the diagnostic criteria guidelines are reviewed and standardized. Moreover, the availability of certain diagnostic criteria is essential to limit the risk of ABPA overdiagnosis. It is likely that due to the fear of lung damage due to ABPA, the diagnosis of this disease is made in only colonized patients who are exposed to unneeded corticosteroid therapy. Further research studies are needed in order to improve *A. fumigatus* diagnosis, define classification of clinical manifestations, as well as personalize the treatment and prevention.

## Figures and Tables

**Table 1:** Laboratory exams at admission and during follow-up.

Blood Exams	Admission	After 6 Days	After 16 Days (Beginning of ABPA Therapy)	After 2 Weeks of ABPA Therapy
Leucocytes, cells/mm^3^	17,130	19,870	17,080	18,060
Neutrophils, %	75.4	66.6	59.3	68.4
Eosinophils, %	2.7	8.0	19.3	5.1
CRP, mg/dL	11.19	16.80	4.26	0.90

ABPA, allergic bronchopulmonary aspergillosis; CRP, C reactive protein.
